# Developing a manually annotated clinical document corpus to identify phenotypic information for inflammatory bowel disease

**DOI:** 10.1186/1471-2105-10-S9-S12

**Published:** 2009-09-17

**Authors:** Brett R South, Shuying Shen, Makoto Jones, Jennifer Garvin, Matthew H Samore, Wendy W Chapman, Adi V Gundlapalli

**Affiliations:** 1 VA Salt Lake City Health Care System, IDEAS Center, Salt Lake City, Utah, 84148, USA; 2 Department of Internal Medicine, University of Utah, Division of Clinical Epidemiology, Salt Lake City, Utah, 84148, USA; 3 Department of Biomedical Informatics, University of Utah, Salt Lake City, Utah, 84148, USA; 4 Department of Biomedical Informatics, University of Pittsburgh, Pittsburgh, PA, USA

## Abstract

**Background:**

Natural Language Processing (NLP) systems can be used for specific Information Extraction (IE) tasks such as extracting phenotypic data from the electronic medical record (EMR). These data are useful for translational research and are often found only in free text clinical notes. A key required step for IE is the manual annotation of clinical corpora and the creation of a reference standard for (1) training and validation tasks and (2) to focus and clarify NLP system requirements. These tasks are time consuming, expensive, and require considerable effort on the part of human reviewers.

**Methods:**

Using a set of clinical documents from the VA EMR for a particular use case of interest we identify specific challenges and present several opportunities for annotation tasks. We demonstrate specific methods using an open source annotation tool, a customized annotation schema, and a corpus of clinical documents for patients known to have a diagnosis of Inflammatory Bowel Disease (IBD). We report clinician annotator agreement at the document, concept, and concept attribute level. We estimate concept yield in terms of annotated concepts within specific note sections and document types.

**Results:**

Annotator agreement at the document level for documents that contained concepts of interest for IBD using estimated Kappa statistic (95% CI) was very high at 0.87 (0.82, 0.93). At the concept level, F-measure ranged from 0.61 to 0.83. However, agreement varied greatly at the specific concept attribute level. For this particular use case (IBD), clinical documents producing the highest concept yield per document included GI clinic notes and primary care notes. Within the various types of notes, the highest concept yield was in sections representing patient assessment and history of presenting illness. Ancillary service documents and family history and plan note sections produced the lowest concept yield.

**Conclusion:**

Challenges include defining and building appropriate annotation schemas, adequately training clinician annotators, and determining the appropriate level of information to be annotated. Opportunities include narrowing the focus of information extraction to use case specific note types and sections, especially in cases where NLP systems will be used to extract information from large repositories of electronic clinical note documents.

## Background

Much of the detailed phenotypic information that is necessary for translational research is only available in clinical note documents and the breadth of clinical information that can be extracted from these documents is profound. Over the last decade researchers have employed a variety of methods ranging from simple keyword based approaches to increasingly complex natural language processing (NLP) systems to extract information from electronic clinical note documents [1-4]. However, significant modifications must be made to customize NLP systems to extract relevant phenotypic and other types of clinical data from different electronic medical record (EMR) systems. In addition, highly templated note documents like those that exist in the US Veteran's Administration Health Care System (VA EMR) pose specific challenges, and at the same time provide opportunities for development of NLP systems used for information extraction (IE) tasks. Equally challenging is to apply annotation methods to build annotated corpora and associated tasks that can be used to build reference standards required for performance evaluation of those systems. Manual annotation tasks are time consuming, expensive, and require considerable effort on the part of human reviewers.

The graphical user interface used at all Veteran's Administration Medical Centers in the US (VA) is called the Computerized Patient Records System (CPRS) and it provides several user tools that allow direct entry of free text information. One such tool, called the Text Integration Utilities (TIU) package, provides concurrent charting functions giving users the ability to electronically enter free text information into a diverse range of clinical report types. VA provider notes may contain free text information entered as traditional narratives. They may also contain copied and pasted sections from other provider note documents, or may contain highly templated note sections. The TIU package also allows providers to create custom pre-compiled documents or template structures that can be modified by individual clinicians or tailored for the operational needs of each hospital or specific VA service [5-7].

Templated clinical notes provide pre-defined section headings that require free text entry of information in a narrative style. In addition, long strings of symptoms may be present that require completion of check boxes, and embedded information such as headers that include patient name and demographics, active medications, vital signs, or laboratory results stored elsewhere in the VA EMR. Templated notes may also contain user defined formatting, additional white space denoting note sections, or other visual cues. It is assumed that the use of highly templated note documents encourages consistent data collection, allows data consistency checks, and aids in the process of order generation, clinician reminders, and communication. Use of templated note documents and standard section headings is one example where structured data collection has been applied to unstructured data sources.

Standardized documentation of clinical encounters focuses on the use of a predefined conceptual flow of note sections and logically ordered methods of recording pertinent patient information. These structures provide a defined method of clinical diagnosis, documenting performance of medical procedures, and follow-up of patient care. These expectations for documentation are established by medical education and training, as well as professional societies, and standards organizations and form the basis for medical communication, coding, billing and reimbursements. More recently with the adoption of the Clinical Document Architecture (CDA) model, the structure and semantics of clinical documentation is being driven towards greater standardization [8].

This pilot project illustrates a practical approach to annotation methods that may aid in information extraction of clinical information from electronic clinical documents. We also sought to demonstrate an open source tool that can be used to conduct annotation of electronic note documents and identify concepts and attributes of interest for a specific clinical use case. Our goal was to build an annotated corpus identifying specific concepts denoting phenotypic, procedural, and medication use information for Inflammatory Bowel Disease (IBD). This includes the complex diseases of Crohn's and ulcerative colitis that have underlying genetic dispositions and are characterized by episodes of exacerbations, and could be considered representative of chronic diseases of interest to translational research. We focus on evaluating the presence of concepts for IBD in specific note sections and document types and demonstrate a practical approach to manual annotation tasks for a specific clinical use case. This approach may reduce the burden of document review when these methods are applied to large clinical data repositories.

## Setting

This project was carried out at the VA Salt Lake City Health Care System in Salt Lake City, Utah which provides care for nearly 40,000 patients in Utah and surrounding states. Each year the VA provides care to almost 6 million veterans with an estimated *638,000 note documents *entered each day at VA facilities nationwide.

## Methods

### Study population and document corpus

In a previous study we conducted a semi-automated review of note documents extracted from the VA EMR using a combination of NLP and string searching coupled with a negation algorithm to identify patients with Inflammatory Bowel Disease (IBD) (n = 91) [9]. For this pilot study we selected the 62 patients from Salt Lake City and a random sample of associated electronic clinical notes for these patients that were generated in a 6-month period (n = 316).

### Operational definitions

**Figure 1:**
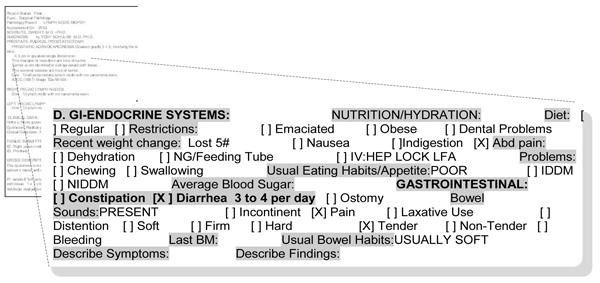
**Templated note sections**. Gray highlights constitute headings and subheadings by our schema. Bolded text indicates the span of templated text that was related to Diarrhea. In this case, Diarrhea is at least dependent on the [] brackets to interpret its presence. **"3 to 4 per day" **represents free text placed in an area that it was not meant to be entered, which depends on **" [X] Diarrhea" **to make sense. In a broader sense, it still relies on its relation to **GASTROINTESTINAL, D. GI-ENDOCRINE SYSTEMS**, BIOPHYSICAL and the instruction clause to give proper context.

**Figure 2:**
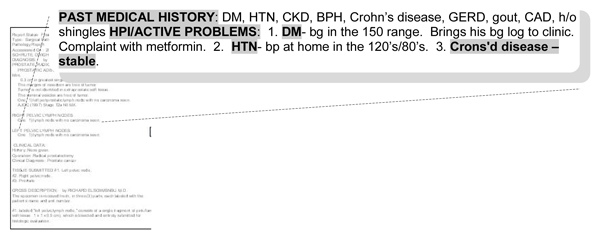
**Predefined headings and subheadings**. By and large, the elements listed here are able to stand on their own below the predefined headings of **"Past Medical History and HPI/Active problems"**. Although this appears to be free-text, an interesting part of this excerpt is that it incorporates dependency structures traditionally used by templates at the subheading level. For example, **"crohns-stable" **relies on its heading to give proper framing.

Medical providers are trained to follow patterns when evaluating patients and writing clinical notes using section headings and note segments. These patterns are important to prevent omission of essential details and capture all necessary data for completeness and billing. We apply an operational definition of note templating and make a distinction between two types of pre-compiled or standardized documentation tools that appear in VA electronic note documents. We provide specific examples of these conditions in Figures 1 and 2.

#### 1) Templated note sections

these are structured note sections that contain check lists and are usually in the form of clinical terms with square brackets, boxes, yes/no pick lists etc. These are usually associated with signs, symptoms and evaluation criteria and are found in documents such as nursing and pre-operative assessments. *The individual elements of a templated section must be included to infer clinical information and can only be interpreted as a complete string in the context of the template *(Figure 1).

#### 2) Pre-defined headings

these denote semi-structured elements and mainly serve as prompts and placeholders for the provider to complete. Examples include chief complaint, history of present illness, medications, laboratory data, etc. *Free text following these headings can stand on its own and be generally interpretable by the reader of the note without the associated heading *(Figure 2).

### Development of the annotation schema and guidelines

**Figure 3:**
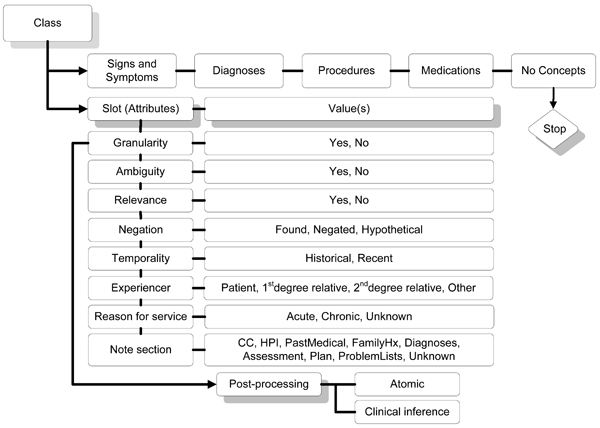
**Knowtator class and slot hierarchy for this annotation task**.

**Figure 4:**
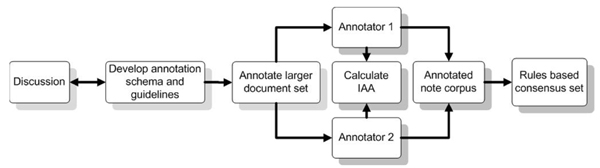
**Process flow diagram for annotation tasks**.

An initial set of annotation guidelines and concept lexicon used for explicit review tasks were developed based on conversations between two internal medicine board-certified physicians (AVG, MJ), informaticians (BRS, SS, WW), and one health information management (HIM) professional (JHG). Based on these same discussions, an annotation schema was developed using an open source knowledge representation system called Protégé [10] and an annotation plug-in tool called Knowtator [11]. Our annotation schema defines four different concept classes including: signs or symptoms, diagnoses, procedures, and medications, and associated concept attributes described below (Figure 3). Over the course of several pilot tests on a small corpus of note documents, the annotation schema and set of guidelines were pilot tested and iteratively refined (Figure 4). We did not create a validation set that could be used for pilot testing or annotator training. However, for large scale annotation tasks where the specific task is complex and the resulting reference standard will be used to train and evaluate performance of NLP systems this step would be advised.

### Annotation of clinical documents

**Figure 5:**
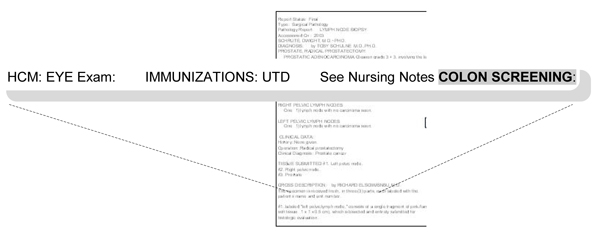
**Concept attribute: relevance**. In this case, we find templated text, with the absence of text after **"colon screening" **probably indicating that the provider either ignored or neglected it, or meant it to be negated. It was a goal concept and thus marked, but colonoscopy probably was not performed and thus the concept is irrelevant in that it does not contribute to the presence or absence of IBD.

Using a final version of the annotation guidelines and schema, we conducted an instance level annotation of the 316 note documents for our sample of IBD patients using Protégé and the Knowtator tool. Two clinician annotators were tasked with identifying and annotating relevant concepts for IBD, using their clinical judgment and an initial lexicon of terms developed representing specific concept classes. For each relevant IBD concept clinician annotators were tasked with indicating the span of text identifying those concepts. Annotators also identified specific concept attributes describing contextual features [12] (Figure 3): 1) negation (found, negated, hypothetical); 2) temporality (historic, recent); 3) patient experiencer (patient, 1^st ^degree relative, 2^nd ^degree relative); 4) reason for service (acute, chronic, unknown); 5) the specific note section in which the concept was found; 5) three concept attributes describing granularity, relevance, and ambiguity [13]. We extend these last three additional properties from the information retrieval [14,15] and terminology literature [16,17] and define them as they were applied to the annotation task as follows:

1) *Concept relevance *– describes how relevant the specific concept is with in the context of the heading or template. *Answers the question: is the concept necessary and relevant for diagnosis given this clinical use case *(Table 1 and Figure 5)?

**Table 1:** Examples of concepts by concept class and concept attributes

	**Granular (atomic)**		**Granular (clinical inference)**		**Relevance**		**Ambiguous**	
**Concept Class**	**Yes**	**No**	**Yes**	**No**	**Yes**	**No**	**Yes**	**No**
**Diagnoses**	Crohn's Disease	pouchitis	Crohn's Disease	Ankylosing Spondylitis	Crohn's Disease	**	UC	Ulcerative Colitis
**Signs and Symptoms**	Diarrhea	flare	**	weight loss	Diarrhea	**	NT	Non-tender
**Procedures**	Colonoscopy	surgery	**	Colectomy	Colonoscopy	EGD	Scope	Colonoscopy
**Medications**	Mesalamine	**	Mesalamine	Steroid	Mesalamine	**	Steroid	Prednisone

** No concept from this use case was identified.

UC = Ulcerative colitis, NT = Non = tender

**Figure 6:**
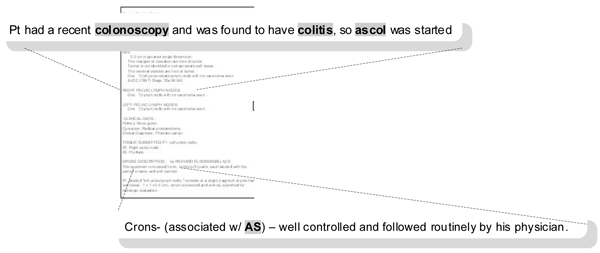
**Concept attribute: ambiguity**. In the top example, **"colitis" **probably represents IBD, but it is certainly not definitive. In the bottom example, although it is inferred that **"AS" **is probably ankylosing spondylitis, the same abbreviation can also be used for aortic stenosis and sclerosis. These are both conditions that are common among older veterans.

*2) Concept ambiguity *– describes the potential for mis-categorization or mis-diagnosis based on how the concept is used in the document. *Answers the question: is the concept ambiguous and would an alternative interpretation lead to mis-categorization or some other diagnosis *(Table 1 and Figure 6)?

**Figure 7:**
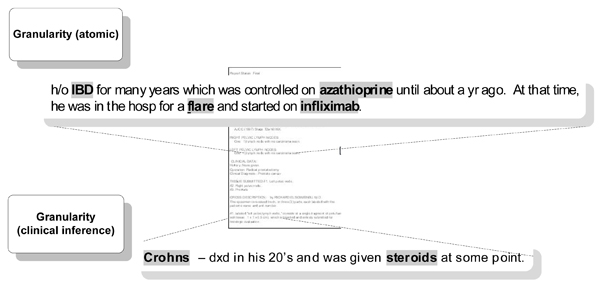
**Concept attribute: granularity**. In the case of Granularity (atomic): **"IBD"**, **"azathioprine"**, and **"infliximab" **would be coded as granular as they were independent at the goal concept level. **"Flare" **is not as we must infer that the provider is talking about a Crohn's disease flare. In the case of Granularity (clinical inference): **"Crohn's" **disease is granular at the level of being able to make a clinical inference of IBD, but **"steroids" **by itself cannot invoke an inference of any particular disease.

*3) Concept granularity *– measures whether the concept is either too generic or specific as it is used. *Answers the question: can the concept stand by itself without need for coordination with other concepts for clinical meaning? *For the annotation task, we defined two levels of granularity: a) the atomic concept level describing whether the mentioned concept stands on its own; and b) the clinical inference level describing whether the concept identified must be coordinated with other concepts to make a clinical diagnosis given our specific use case (Table 1 and Figure 7).

### Developing a rules-based consensus set

We reviewed disagreements identified from the completed and merged clinician annotation projects derived from the annotation task. We then developed specific rules to build a consensus set that we could apply programmatically using the following use case specific logic: 1) We selected annotations where spans from each annotator overlap and attributes have the same values; 2) In the case where annotation spans overlapped, but were not identical we selected for the shorter span; 3) We preserved concepts where one reviewer identified the concept and the other did not; 4) In instances where annotations overlapped, but there was disagreement at the attribute level, we retained the values selected by the senior physician annotator.

### Annotator agreement and levels of evaluation

We estimate agreement between the two annotators for specific annotation tasks as described by Hripcsak [18,19] and Roberts [20], using Cohen's Kappa where true negatives were available and F-measure otherwise. We also report the distribution of concepts by concept class and specific attribute, clinical document type, and note section.

## Results

The note corpus corresponding with the patient encounters selected for this pilot study included 316 notes with 92 unique note titles. We classified note documents into the following categories: primary care associated including new and established patient visits (40%), ancillary services for occupational therapy, nutrition and short addenda (31%), specialty clinic including the Gastro-intestinal (GI) clinic (15%), emergency department (8%) and peri-procedure related notes (6%). Clinician annotators completed a total number of 1,046 annotations related to our specific use case (IBD) that included annotations for concepts indicating signs and symptoms (395, 38%), diagnoses (249, 24%), procedures (239, 23%), and medications (163, 15%). The annotation task took a total of 28 hours with each annotation requiring an average of 50 seconds to identify a concept and associated attributes.

### Annotator agreement estimates

At the document level, clinician annotator agreement (with 95% CI) on whether the documents contained relevant concepts was high at 0.87 (0.82, 0.93). At the concept class level, clinician annotator agreement was highest for the diagnoses concept class (0.83) and lowest for the signs and symptoms concept class (0.61). Agreement over all concept classes was 0.72. Ascertaining the context of specific concept attributes proved to be a more difficult task for clinician annotators as compared to the level of document classification and concept class identification. The only exception was in assessing the experiencer concept attribute (kappa = 1.00), where all but one concept was annotated as describing the patient, as opposed to patient relatives or proxies. Agreement for the relevance concept attribute could not be calculated as one annotator marked all selected concepts as relevant. For the remaining concept attributes, kappa ranged from 0.67 (0.60, 0.74) for negation to 0.06 (0.03, 0.09) for reason for service (Table 2).

**Table 2:** Estimated agreement across various levels of analysis

**Unit of Analysis**	**Kappa (95% CI)**	**F-measure**
**Document**	0.87 (0.82,0.93)	
**Concept**		
Signs and Symptoms		0.61 (0.57, 0.86)
Diagnoses		0.83 (0.80, 0.87)
Procedures		0.63 (0.56, 0.68)
Medications		0.82 (0.76, 0.86)
all classes		0.72 (0.70, 0.74)
**Attribute**		
Granularity	0.34 (0.28,0.41)	
Ambiguity	0.08 (0.04,0.13)	
Relevance	**	
Negation	0.67 (0.60,0.74)	
Temporality	0.67 (0.61,0.73)	
Experiencer	1	
Reason for Service	0.06 (0.03,0.09)	
Note Section	0.54 (0.50,0.59)	

** Kappa for relevance could not be estimated

*** Only 1 concept was annotated as describing an experiencer other than the patient.

### Concept and concept attribute level analysis

We calculated the average number of annotated concepts per document, stratified by document category and concept type (Table 3). This estimate was used to represent the yield of annotations per document. Not surprisingly, GI clinic notes produced the highest yield per document for all 4 types of concepts, ranging from 1.7 procedure-related concepts to 3.8 signs and symptoms related concepts per document. Primary care notes provided the second highest yield for concepts indicating diagnoses, procedures and medications, while emergency department notes provided the second highest yield for average number of concepts for signs and symptoms. The lowest yield for IBD relevant concepts was for ancillary service notes which include short addenda to main notes, chaplain service notes, etc. Although ancillary service notes made up 31% of the document corpus, only 37 (4%) concepts associated with our use case were identified within these documents.

**Table 3:** Yield of concept classes by document type

	**Annotated Concepts per Document (# concepts)**				
**Document type**	**Clinical Documents**	**Diagnoses**	**Signs and Symptoms**	**Procedures**	**Medications**
**Ancillary Services**	**98**	0.1 (12)	0.1 (12)	0.04 (4)	0.09 (9)
**Emergency Note**	**24**	0.7 (17)	2.2 (53)	0.2 (4)	0.7 (16)
**Peri-procedure**	**19**	0.3 (6)	0.9 (18)	0.2 (3)	0.1 (2)
**Primary Care**	**127**	1.4 (172)	2.0 (251)	1.6 (204)	0.7 (92)
**Specialty Clinic**	**47**	0.8 (37)	1.2 (57)	0.5 (22)	0.9 (41)
GI Clinic	**10**	**2.1 (21)**	**3.6 (36)**	**1.7 (17)**	**2.8 (10)**
Other Specialty Clinic	**37**	0.4 (16)	0.6 (21)	0.1 (5)	0.4 (13)

In addition, we also examined the occurrence of concepts annotated *within different sections of the clinical documents*. Major note sections where clinicians annotated concepts included assessment, chief complaint, family history, health care maintenance (HCM), history of presenting illness (HPI), medications, past medical history, plan, problem lists, review of systems, and physical examination. Of these sections, assessment contained the majority of annotated concepts (171, 16.3%), with the HPI section following closely (167, 16.0%). Family history and plan sections contained the least numbers of annotated concepts, having 1 (0.1%) and 9 (0.9%) concepts respectively.

We then calculated the prevalence of each annotated concept class in the top 2 most frequent sections it appeared in, as well as the attributes of the annotated concepts in terms of being ambiguous, relevant to IBD, granular at the atomic level, and granular at the clinical inference level (Table 4). Over two-thirds (72%) of annotated terms used for signs and symptoms were identified as being ambiguous. Clinician annotators selected only 18 (2%) terms representing medications they believed were ambiguous with reference to goal IBD concepts. Most of the concept ambiguity identified by clinician annotators resulted from use of abbreviations, synonyms, as well as use of concepts that require post-coordination to make clinical inferences. Though not quantified, there were instances of boxes and checklists "unchecked" that resulted in ambiguity.

**Table 4:** Concept classes and note sections by affirmed concept attributes

**Concept Class**	**Concepts**	**Ambiguous**	**Relevant**	**Granular (atomic)**	**Granular (clinical inference)**
**Diagnoses**	**249**	46 (18%)	**249 (100%)**	245 (98%)	192 (77%)
Assessment	**68 (27%)**	13 (5%)	68 (27%)	67 (27%)	53 (21%)
Problem Lists	**56 (22%)**	14 (6%)	56 (22%)	55 (22%)	40 (16%)
**Signs and Symptoms**	**395**	283 (72%)	391 (99%)	257 (65%)	**0**
HPI	**91 (23%)**	58 (15%)	89 (23%)	66(17%)	
Physical Examination	**81(21%)**	65 (16%)	80 20%)	27 (7%)	
**Procedures**	**239**	116 (49%)	226 (95%)	207 (87%)	**0**
HCM	**55 (23%)**	29 (12%)	55 (23%)	55 (23%)	
Assessment	**34 (14%)**	18 (8%)	33 (14%)	27 (11%)	
**Medications**	**163**	18 (11%)	157 (96%)	**163 (100%)**	133 (82%)
Medication	**64 (39%)**	4 (2%)	61 (37%)	64 (39%)	48 (29%)
Assessment	**37 (27%)**	5 (3%)	36 (22%)	37 (27%)	30 (18%)

**Total**	**1046**	463 (44%)	1023 (98%)	872 (83%)	325 (31%)

HPI = History of presenting illness, HCM = Health Care Maintenance

All annotated medications, and the majority of annotated diagnoses (98%), procedures (87%), and signs and symptoms (65%) were deemed granular at the atomic level *(concept stands on its own)*. However none of the identified concepts denoting signs and symptoms were believed granular enough at the level of clinical inference for IBD. On the other hand, clinician reviewers determined that most annotated medications (82%) and diagnoses (77%) were granular at the clinical inference level. Over 95% of annotated concepts were considered relevant to IBD due to the fact that the notes were drawn from encounters of patients known to have IBD.

Annotators also identified specific attributes describing contextual features for concept negation, temporality, and experiencer (Table 5). The majority of concepts denoting signs and symptoms (61%) were found to be negated. Reason for service could not be ascertained for 98% of all annotated concepts for diagnoses. The majority of concepts for signs and symptoms (66%) were associated with concepts describing acute conditions, whereas the majority of procedures (60%) were associated with concepts describing chronic conditions. Finally, in our random sample of notes, an experiencer other than the patient was identified in only 1 out of 249 (0.4%) annotated diagnoses and in none of the other concept classes. This last finding has important implications for translational research particularly for conditions like Crohn's disease known to have a genetic component.

**Table 5:** Distribution of contextual attributes by concept classes

**Attribute**	**Diagnoses**	**Signs and Symptoms**	**Procedures**	**Medications**
**Negation**				
found	239 (96%)	130 (34%)	201 (84%)	152 (94%)
negated	3 (1%)	**242 (61%)**	15 (6%)	3 (2%)
hypothetical	7 (3%)	22 (5%)	23 (10%)	6 (4%)
**Temporality**				
historic	236 (95%)	87 (22%)	176 (74%)	70 (43%)
recent	13 (5%)	307 (78%)	63 (26%)	93 (57%)
**Reason for service**				
acute	4 (1.6%)	**262 (66%)**	81 (34%)	103 (63%)
chronic	1 (0.4%)	119 (30%)	144 (60%)	55 (34%)
unknown	**0 (0%)**	1 (<0.1%)	1 (<0.1%)	4 (2%)
**Experiencer**				
patient	248 (99.6%)	395 (100%)	239 (100%)	163 (100%)
1st degree relative	**1 (0.4%)**	0	0	0
2nd degree relative	0	0	0	0
other	0	0	0	0

**Total**	**249**	**395**	**239**	**163**

## Discussion

We have identified specific challenges and opportunities posed by highly templated clinical note documents including identifying note types or sections that will provide the highest concept yield, and adequately training NLP systems to accurately process templated note sections. "Unchecked" boxes in checklists also pose a dilemma for clinical inferencing. Depending on the clinical question, resources could be directed to process and review those note types with the highest expected yield. Moreover, other types of information could certainly be extracted from clinical narratives besides those in our annotation schema. Also algorithmic approaches could be developed and applied to identify specific note sections and templated note structures. There may also be opportunities to code section headings and template types using the UMLS or a terminology such as SNOMED-CT that allows coordination of concepts. Note sections could also be extracted in a standardized format using the HL7 CDA model.

Our results and conclusions are drawn from data representing an example of only one chronic disease. We purposefully selected documents from patients known to have IBD and did not review documents for patients not known to have IBD. We arrived at a rules-based consensus set that was derived by looking at a subset of note documents containing the highest number of concepts. This was a practical approach considering the duration of time required for clinician annotators to individually annotate the full corpus of 316 documents.

There is also an implied need to add a measure of uncertainty to our annotation schema since agreement was low at the concept attribute level. Additionally, it is necessary to conduct rigorous and adequate discussions of the lexicon used for and common interpretations and definitions of how concept attributes are to be applied prior to and during annotation tasks [11,19,21]. It became evident that clinicians over the course of the annotation task used an evolving understanding of our annotation schema and developed internal definitions that may have drifted over time. We could not quantify this drift given our study design and data from the resulting annotated corpus.

## Conclusion

The results of this pilot study will inform further work at the VA, where major efforts are underway to build annotated corpora and apply NLP methods to large data repositories. We provide an example of a fairly complex annotation schema applied to highly templated note documents. When confronted with a large data repository of electronic clinical documents, it is likely that it is only necessary to apply IE tools on certain note types and/or note sections to identify phenotypic information useful for translational research. However, defining specific information to be annotated depends on the clinical questions asked and at what level one wishes to extract information from clinical text.

These methods could be expanded to further enhance medical terminologies with the goal of building ontologic representations and knowledge bases for specific medical domains. Active learning methods could also be applied to combine the tasks of expert human annotation and training of NLP systems. Finally, we propose that the CDA could be used to identify specific note types and sections to reduce the burden of searching notes for relevant clinical question dependent information.

## Competing interests

The authors declare that they have no competing interests.

## Authors' contributions

BRS conceived the study based on initial discussions with SS and AVG, helped develop guidelines and annotation schema, and wrote all drafts of the manuscript. SS participated in design and construction of the annotation schema, and provided statistical analyses of data derived from annotation efforts. MJ annotated all clinical documents and helped with annotation schema, guideline development and study design. JHG participated in initial design of annotation schema and guidelines, and manuscript preparation. MHS provided funding support and facilities for this study and participated in study design. WC helped with annotation schema and guidelines. AVG annotated all study clinical documents and provided overall oversight and guidance for the study. All authors read drafts and approved the final manuscript.
